# Rheumatoid arthritis synovial fibroblasts modulate T cell activation

**DOI:** 10.1172/jci.insight.193054

**Published:** 2025-10-07

**Authors:** Melissa R. Romoff, Preethi K. Periyakoil, Edward F. DiCarlo, Daniel Ramirez, Susan M. Goodman, Christina S. Leslie, Alexander Y. Rudensky, Laura T. Donlin, Melanie H. Smith

**Affiliations:** 1 Research Institute, Hospital for Special Surgery, New York, New York, USA.; 2 Computational and Systems Biology Program, Memorial Sloan Kettering Cancer Center, New York, New York, USA.; 3 Department of Pathology and Laboratory Medicine, and; 4 Division of Rheumatology, Department of Medicine, Hospital for Special Surgery, New York, New York, USA.; 5 Weill Cornell Medical College, New York, New York, USA.; 6 Howard Hughes Medical Institute and Immunology Program at Sloan Kettering Institute, Ludwig Center for Cancer Immunotherapy, Memorial Sloan Kettering Cancer Center, New York, New York, USA.

**Keywords:** Autoimmunity, Immunology, Rheumatology

## Abstract

In the rheumatoid arthritis (RA) synovium, resident fibroblast-like synoviocytes (FLS) express MHC class II molecules (HLA-D) but lack the costimulatory signals typically required for T cell activation. Here, we demonstrate that antigen presentation by FLS induces a distinct T cell activation state characterized by high CD69 yet reduced CD25 and HLA-DR expression, suppressed proliferation, and decreased effector cytokine production compared with professional antigen-presenting cells (APCs), such as macrophages. FLS were also capable of suppressing macrophage-induced T cell activation, underscoring their dominant immunomodulatory role in the synovial microenvironment. Mechanistically, we identify indoleamine 2,3-dioxygenase–mediated (IDO1-mediated) tryptophan depletion as the primary driver of FLS-induced T cell hyporesponsiveness. Spatial transcriptomics revealed colocalization of *IDO1* and *CD69* within ectopic lymphoid structures in RA synovium, further supporting the in vivo relevance of this pathway. These findings provide the groundwork for positioning FLS as critical T cell regulators in RA and highlight the importance of preserving their immunosuppressive properties when therapeutically targeting pathogenic FLS functions.

## Abstract 2

Joint-resident fibroblasts in rheumatoid arthritis engage T cells via their T cell receptor (TCR) and induce T cell hypo-responsiveness.

## Graphical abstract

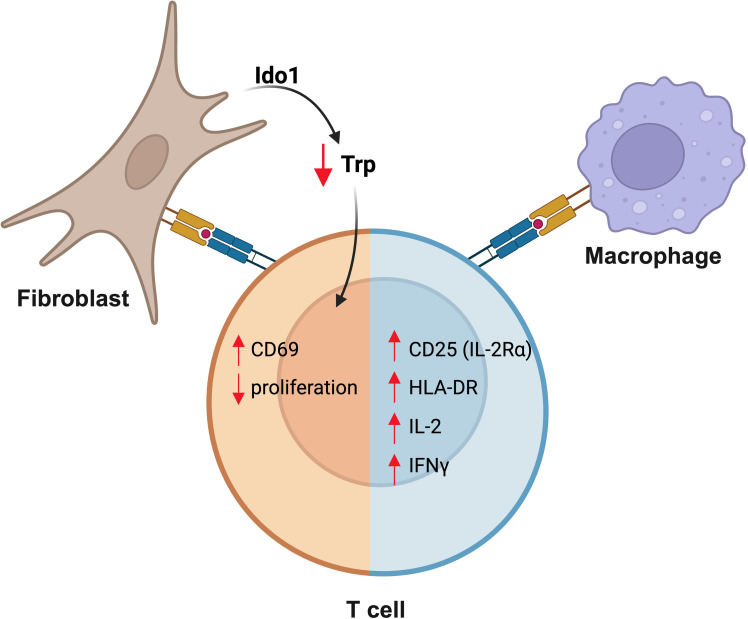

## Introduction

In healthy joints, the synovial lining (synovium) primarily consists of stromal cells, of which synovial fibroblasts, also known as fibroblast-like synoviocytes (FLS), are one of the most abundant. In autoimmune inflammatory arthritis, such as rheumatoid arthritis (RA), blood-derived leukocytes infiltrate the synovium, altering both the phenotypes and activation states of resident FLS. In RA, the sublining FLS expand into multiple subsets with distinct predicted functions (1–3). Among these, one population is characterized by a gene signature responsive to cytokines IFN-γ and TNF, which includes expression of MHC class II (HLA-D) (4). FLS HLA-D expression is notably widespread in the inflamed synovium, with higher levels observed in leukocyte-rich as compared with leukocyte-poor RA (1, 5). This upregulation is likely driven by IFN-γ produced by infiltrating T and NK cells in close proximity to sublining FLS (1, 5). Additionally, FLS within lymphocyte aggregates, or ectopic lymphoid structures (ELS), exhibit nuclear localization of phosphorylated STAT1, indicating active signaling in response to IFN-γ (4).

While the role of HLA-D expression by FLS in vivo remains unclear, several studies have demonstrated the capacity of FLS to present antigens to T cells in vitro (5–9). Although FLS lack costimulatory molecules (10), presentation of superantigen staphylococcal enterotoxin B (SEB) by HLA-DR molecules expressed by FLS can activate CD4^+^ T cells as evidenced by expression of the activation marker CD69 (5). Moreover, FLS can present peptide antigens bound to HLA-DR molecules to corresponding antigen-specific T cells, leading to IL-2 expression (7, 8). While this work has shown that FLS are capable of inducing CD69 and IL-2 expression by T cells through the antigen presentation by MHC class II molecules, it remains unclear how their ability to activate T cells compares with a professional antigen-presenting cell (APC), such as macrophages and dendritic cells (DCs).

In this study, we perform a comparative analysis of T cell activation states induced by antigen presented by FLS versus professional APCs. Our findings suggest that FLS, functioning as APCs, limit T cell activation and that FLS play an immunomodulatory role within the inflamed synovium.

## Results

### FLS and macrophages drive distinct T cell activation states.

Using either primary FLS or monocyte-differentiated macrophages as APCs, we examined T cell activation in response to the superantigen SEB. We measured blood-derived T cell activation using flow cytometric analysis of canonical markers of T cell activation: CD69, CD25 (IL-2 receptor α chain; IL-2RA), and HLA-DR. As SEB selectively activates only specific Vβ element–expressing TCRs (11), we were able to distinguish between those T cells that were stimulated directly by SEB through TCR cross-linking (Vβ^SEB^: pooled Vβ3, 13.2, 14, and 17) versus as bystanders (Vβ^non^: pooled Vβ2 and 5.1).

**Figure 1:**
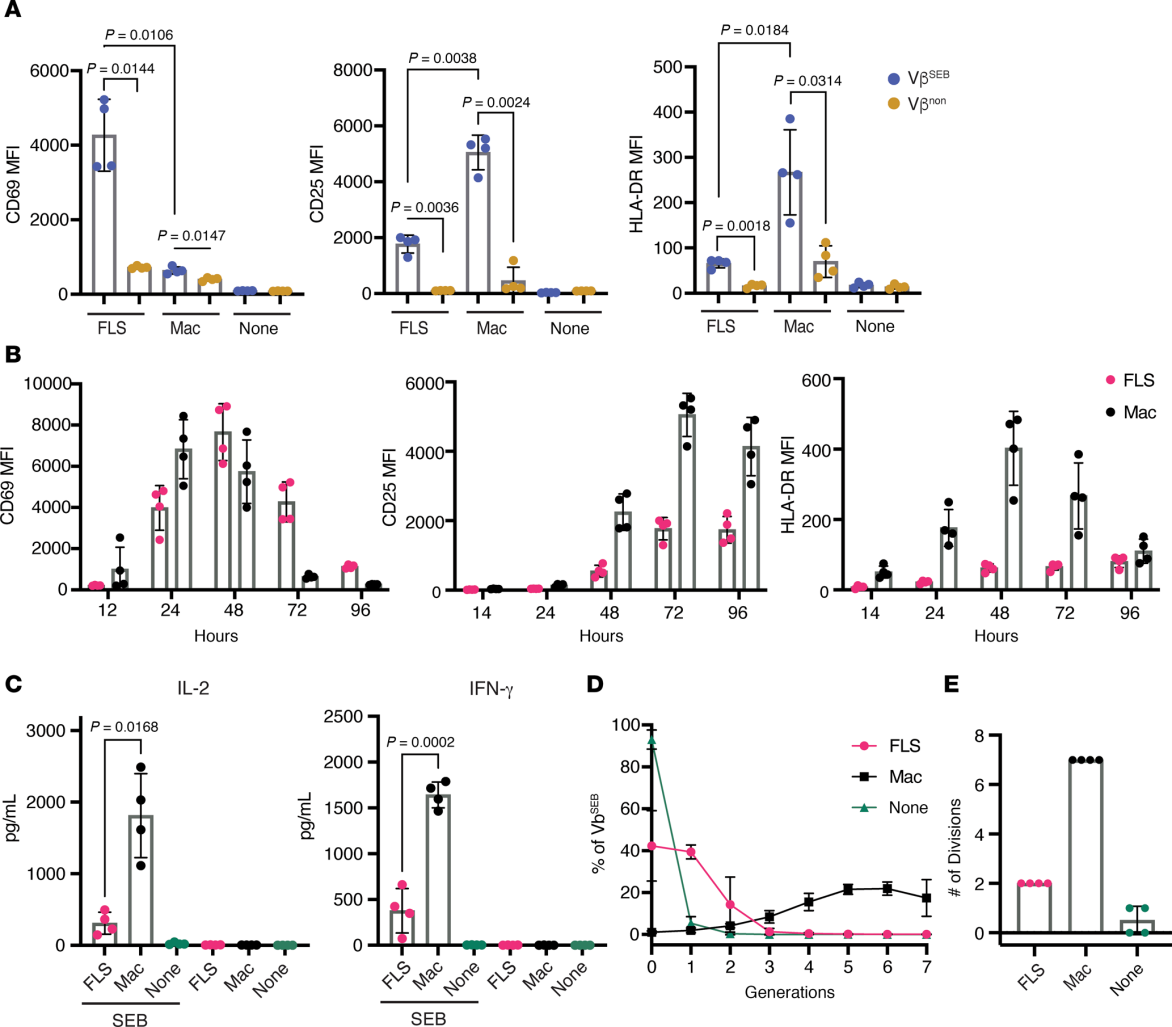
Antigen presentation by FLS results in altered T cell activation characterized by exaggerated and prolonged CD69 expression. Primary RA FLS or blood-derived M-CSF differentiated macrophages were used as APCs of SEB. Autologous macrophages and T cells were isolated from healthy donor PBMCs, and SEB was added to all conditions. (**A**) Surface expression of T cell activation markers CD69, CD25, and HLA-DR was measured via flow cytometry as mean fluorescence intensity (MFI) after 72 hours of SEB activation. The *x* axis indicated the APC type, where “none” denotes the absence of APCs. Data points are color-coded by T cell Vβ sequencing: Vβ^SEB^ indicates T cells with SEB-reactive Vβ sequences (pooled Vβ 3, 13.2, 14, 17), and Vβ^non^ represents T cells with nonreactive Vβ sequences (pooled Vβ 2, 5.1). *N* = 4. Mean ± standard deviation is shown. Statistical comparisons were made using paired 2-tailed *t* tests with corrections for multiple comparisons within each outcome to maintain an overall type I error rate 0.05 as described in the methods section. (**B**) MFI of CD69, CD25, and HLA-DR on CD3^+^ Vβ^SEB^ T cells over the course of 96 hours. Magenta: T cells with FLS as APCs; black: T cells with macrophages as APCs. *N* = 4. (**C**) Cytokine concentrations measured by Luminex in the supernatant of APC–T cell cocultures with or without SEB stimulation for 48 hours. Statistical comparisons made using paired 2-tailed *t* tests. *N* = 4. (**D**) Proliferation of CD3^+^ Vβ^SEB^ T cells after 6 days of SEB stimulation. Percentage of CD3^+^Vβ^SEB^ T cells in each of 7 observed generations (divisions). (**E**) Maximal number of divisions with ≥5% CD3^+^Vβ^SEB^ T cells. For **D** and **E**, magenta: T cells with FLS as APCs; black: T cells with macrophages as APCs; teal: no APC. *N* = 4.

We observed that T cells directly activated through SEB-induced TCR cross-linking (Vβ^SEB^) exhibited higher expression of activation makers than their non-SEB-reactive counterparts (Vβ^non^) (Figure 1A and Supplemental Figure 1A; supplemental material available online with this article; https://doi.org/10.1172/jci.insight.193054DS1). Compared with macrophages, FLS induced higher CD69, but lower CD25 and HLA-DR expression, on the T cell surface after 72 hours of SEB-induced activation (Figure 1A). Although the difference in activation marker expression between SEB-reactive (Vβ^SEB^) and nonreactive (Vβ^non^) T cells indicates HLA-D/TCR-dependent activation, we confirmed that both CD69 expression driven by FLS and CD25 expression driven by macrophages is HLA-DR dependent using a blocking antibody (Supplemental Figure 1B). The CD69 expression driven by FLS was primarily observed on conventional CD4^+^ T cells, with no significant expansion of Foxp3^+^ regulatory T cells in the presence of FLS (Supplemental Figure 1, C–E).

Consistent with our findings using healthy donor T cells, synovial T cells expressed higher levels of CD69 and lower levels of CD25 when FLS were used as APCs compared with autologous macrophages differentiated from patient PBMCs (Supplemental Figure 2A). We performed cocultures using T cells, FLS, and macrophages, all isolated from RA synovium. Although there was a wide range of CD69 expression prior to culture, after 72 hours of SEB stimulation, T cells cultured with FLS consistently displayed higher CD69 and lower CD25 and HLA-DR compared with those cultures with macrophages (Supplemental Figure 2B). As RA synovial cells results paralleled those of healthy donors, and the number of T cells and macrophages obtainable from RA tissue was limited, subsequent experiments were performed with healthy donor leukocytes.

To better understand the differences in T cell dynamics induced by the 2 APC types, we conducted a time course experiment over 96 hours. While macrophages induced higher transient CD69 expression on T cells at early time points (12 and 24 hours), FLS-driven CD69 expression on T cells surpassed macrophages at later time points (72 and 96 hours) (Figure 1B). This exaggerated and prolonged CD69 expression by T cells in response to FLS was limited to CD69, as macrophages consistently induced higher CD25 and HLA-DR expression by T cells across all time points (Figure 1B). Along with these functional T cell activation markers, CD25 (IL-2RA) and HLA-DR, T cells produced more IL-2 and IFN-γ with macrophages as APCs (Figure 1C). At later time points, macrophages also induced increased CD4 expression, a feature not observed with FLS, supporting global differences in the activation profiles of T cells stimulated by these APCs (Supplemental Figure 2C). In line with previous studies and supporting global differences in T cell activation, we observed that FLS limited T cell proliferation compared with macrophages (Figure 1, D and E).

The prolonged CD69 expression by T cells induced by SEB presented by FLS prompted us to investigate differences in APC–T cell interaction dynamics. Live cell imaging revealed that FLS maintained longer average contacts with T cells compared with macrophages and showed a nonsignificant trend toward fewer contacts per FLS (Supplemental Figure 3). While prolonged T cell–APC contact is generally considered conducive to T cell activation, this does not appear to be the case with FLS as APCs. FLS may instead function to restrict T cell migration by driving exaggerated CD69 expression (12).

**Figure 2:**
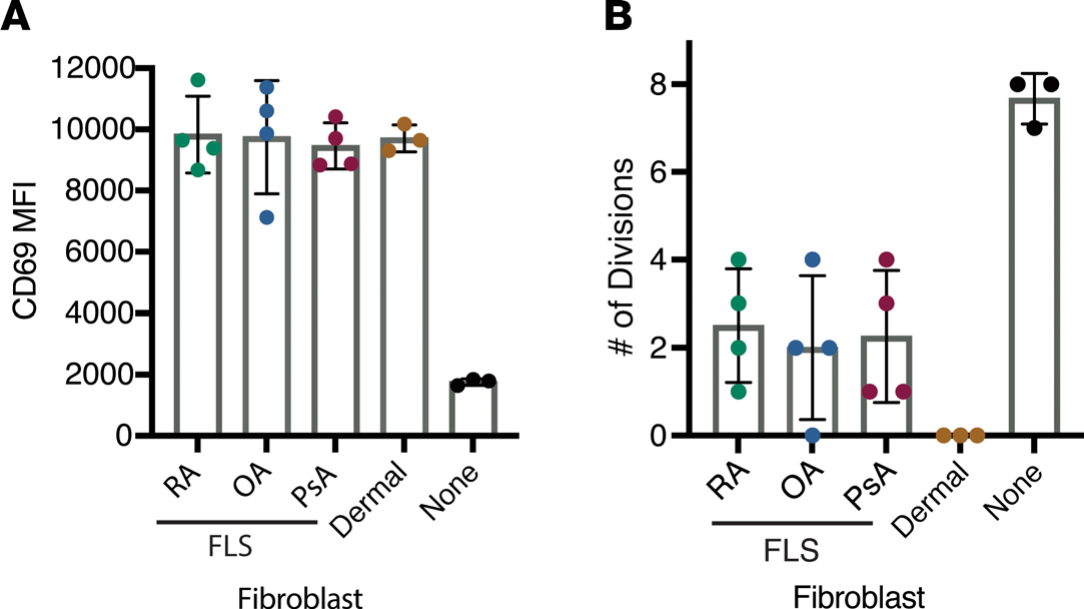
Immunomodulatory features are shared by FLS from rheumatoid, psoriatic, and osteoarthritis samples. CD3^+^ T cells activated with anti-CD3/CD28 beads in the presence of primary FLS from 4 donors each from patients with RA, osteoarthritis (OA), or psoriatic arthritis (PsA) as well as a dermal foreskin fibroblast cell line (Hs27) for 6 days. T cells activated alone served as a control (none). FLS from RA and OA patients were isolated from synovial tissue with a high degree of lymphocytic inflammation by histological scoring. CD3^+^ T cells from the same healthy donor were used with each of the fibroblast conditions. (**A**) CD69 expression on the T cell surface. (**B**) Proliferation measured by CellTrace Violet intensity via flow cytometry. Maximal number of divisions with ≥5% of CD3^+^ T cells is shown. For both **A** and **B**, individual data points in the FLS conditions indicate different primary FLS donors (*N* = 4). The 3 data points from the dermal fibroblast and no fibroblast condition (none) are technical replicates (*N* = 3).

Prior studies have indicated that RA FLS have a decreased capacity to suppress T cell proliferation as compared with osteoarthritis (OA) FLS (13). Therefore, we compared the ability of synovial fibroblasts from patients with RA, OA, and psoriatic arthritis (PsA) as well as a dermal fibroblast line (Hs27) to support T cell CD69 expression and T cell proliferation. We found that FLS from patients with all 3 diseases, as well as the dermal fibroblasts, induced enhanced CD69 expression by T cells while suppressing their proliferation to a similar degree (Figure 2 and Supplemental Figure 4). Notably, proliferation was completely absent in dermal fibroblast cocultures, suggesting that the observed effects may not be unique to FLS. While the extreme suppression by the dermal cell line may be due to heightened responsiveness to IFN-γ, additional work will be needed to understand mechanistic differences between fibroblasts of different origins.

### FLS induce a distinct T cell activation program.

**Figure 3:**
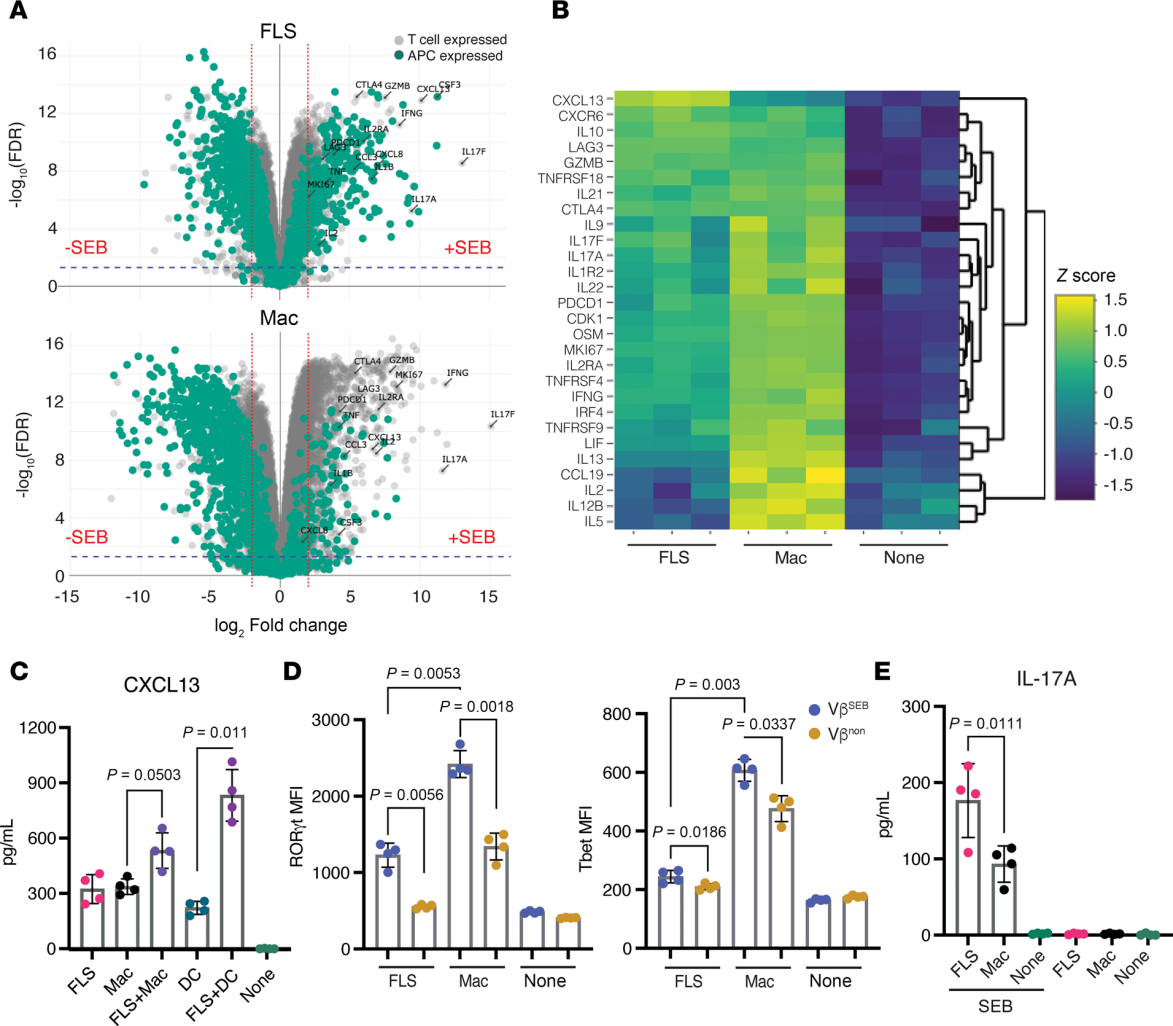
Distinct transcriptional and functional T cell responses to antigen presentation by FLS. (**A**) Volcano plots of differentially expressed genes by bulk RNA sequencing in APC–T cell cocultures with or without SEB after 48 hours (FLS: top, macrophage: bottom). Genes colored in teal are primarily expressed by the APCs. Genes colored in gray are primarily expressed by T cells. (**B**) Heatmap of T cell–expressed genes of interest across conditions (FLS, macrophages, none). *N* = 3. (**C**) CXCL13 concentration in supernatants of APC–T cell cocultures with SEB stimulation for 48 hours. APC identity shown on the *x* axis. *N* = 4. (**D**) RORγt and T-bet expression (MFI) as measured via intracellular transcription factor staining after 72 hours of SEB activation. The *x* axis indicated the APC type. Data points are color-coded by T cell Vβ SEB responsiveness as in Figure 1A. *N* = 4. (**E**) IL-17A concentration in supernatants of APC–T cell cocultures with SEB stimulation for 48 hours. APC identity shown on the *x* axis. *N* = 4. For **C**–**E**, mean ± standard deviation is shown, and statistical comparisons were made using paired 2-tailed *t* tests with corrections for multiple comparisons within each outcome to maintain an overall type I error rate 0.05 as described in the Methods section.

To understand global differences in T cell activation between FLS and macrophages as APCs, we performed bulk RNA sequencing of cocultures after 48 hours of SEB activation. We compared SEB-activated APC–T cell cocultures with those without SEB stimulation (Figure 3A). To identify genes primarily expressed by the APCs, we cultured APCs in the bottom chamber of a Transwell while T cells were activated with anti-CD3/CD28 beads in the insert above.

Analysis of differentially expressed genes revealed that a greater proportion of SEB-induced genes in FLS–T cell cocultures were expressed by the FLS, whereas macrophage–T cell cocultures showed more SEB-induced genes originating from T cells (Figure 3A). When comparing the expression of specific genes of interest across conditions (FLS, macrophages, or no APCs), transcripts of the effector cytokine *IFNG* as well as other cytokines (*IL5*, *IL13*) were more highly expressed with macrophages as APCs (Figure 3B and Supplemental Table 1) in agreement with our protein expression data for IFN-γ (Figure 1C). Consistent with prior findings regarding FLS suppression of T cell proliferation, cell cycle genes (*CDK1*), the proliferation marker gene *MKI67*, and *IL2*, which is needed for T cell growth, proliferation, and differentiation, were all more highly expressed in the condition with macrophages as APCs. Interestingly, *CXCL13* was the most differentially expressed gene in T cells activated with FLS as APCs, suggesting a potential role for FLS in promoting T cell differentiation toward a peripheral or follicular helper phenotype. This was supported by higher expression of *BCL6* and *MAF* in T cells with FLS as APCs (Supplemental Figure 5A). When we assessed soluble CXCL13 levels in the supernatant, they were equivalent with FLS versus macrophages as APCs. However, FLS cocultured with either macrophages or DCs as APC increased CXCL13 expression with only the coculture with DCs resulting in a significant difference (Figure 3C). The discrepancy between *CXCL13* mRNA and protein expression data may be due to the higher number of professional APCs necessary for experiments involving differentiation of DCs (see Methods). Genes highly expressed by FLS during SEB stimulation included *CSF3* (granulocyte colony-stimulating factor) and *CXCL8* (IL-8), suggesting that antigen presentation by FLS may be associated with T cell–dependent support for neutrophil survival, maturation, and chemotaxis.

To understand the effect of FLS versus macrophages on T helper cell differentiation, we performed intracellular cytokine staining for key transcription factors: RORγt (*RORC*) and T-bet (*TBX21*) (Figure 3D). Consistent with RNA-sequencing results, T cells activated by macrophages expressed more of both RORγt, which regulates Th17 differentiation, and T-bet, which regulates Th1 differentiation and promotes IFN-γ expression (Figure 3D and Supplemental Figure 5A). However, IL-17A levels were higher with FLS as APCs (Figure 3E), differing from the gene expression data, indicating differences between transcriptional and translational regulation in this setting. In addition, it has previously been reported that RORγt and IL-17 expression are not as tightly linked in human as in murine cells (14), and IL-17 expression may be modulated through posttranslational modification of RORγt (15).

### FLS antigen presentation to memory T cells.

**Figure 4:**
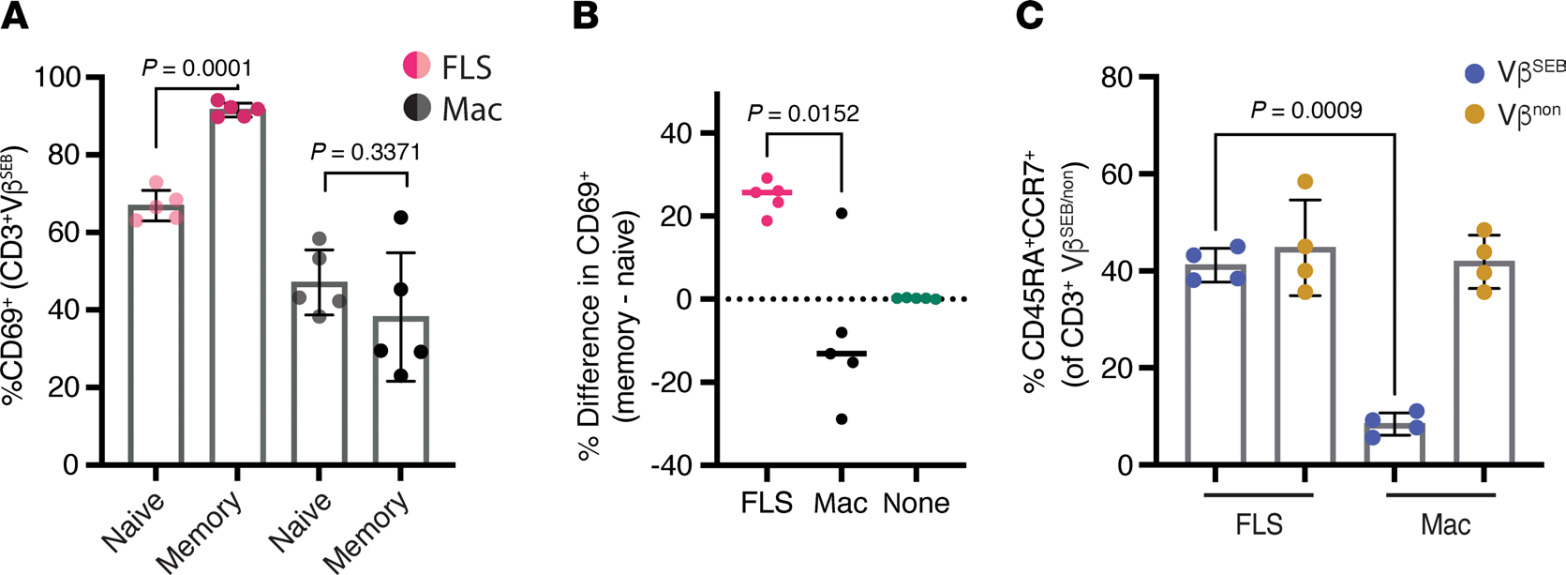
FLS exert a greater effect on memory T cells. (**A**) Sorted naive (CD45RA^+^CCR7^+^) and memory (CD45RA^–^) CD3^+^ T cells from healthy donor PBMCs were cultured with FLS or macrophages as APCs and stimulated with SEB for 72 hours. The percentage of CD69^+^ cells of the CD3^+^ Vβ^SEB^ T cells with FLS (magenta/pink) versus macrophage (black/gray) as APC was determined based on starting T cell population (naive versus memory). *N* = 5. (**B**) Using data from **A**, the percentage difference between the memory and naive %CD69^+^. The difference with no APC present (none) is shown as a control. (**C**) The percentage of CD45RA^+^CCR7^+^ naive T cells with FLS or macrophages as APCs after stimulation with SEB for 72 hours. *N* = 4. For all panels, mean ± standard deviation is shown, and statistical comparisons were made using paired 2-tailed *t* tests.

As most T cells in the joint exhibit a memory phenotype (16, 17), we hypothesized that FLS preferentially modulate the activation of memory T cells over the naive T cells typically found in the periphery. To test this, we sorted naive (CD45RA^+^CCR7^+^) and memory (CD45RA^–^) CD3^+^ T cells from healthy donor PBMCs (Supplemental Figure 5B) and separately stimulated them with SEB for 72 hours using FLS or macrophages as APCs. With FLS as APCs, a higher percentage of memory Vβ^SEB^ T cells expressed CD69 as compared with naive Vβ^SEB^ T cells (Figure 4A and Supplemental Figure 5C). In contrast, no difference was observed in the percentage of Vβ^SEB^ T cells expressing CD69 between naive and memory T cells when macrophages were used as APCs. Quantification of the percentage difference in Vβ^SEB^CD69^+^ expression between memory and naive T cells revealed a significantly larger positive difference with FLS as APCs (Figure 4B). We also found that macrophages, but not FLS, selectively decreased the percentage of CD45RA^+^CCR7^+^ naive cells in SEB-reactive T cells (Figure 4C). The inability of FLS to activate naive T cells and convert them to a memory phenotype is expected, as they do not express costimulatory molecules CD80/CD86 (18). Given that most synovial T cells exhibit a memory phenotype, these results agree with our findings that FLS drive high T cell CD69 expression in T cells isolated from the synovium (Supplemental Figure 2, A and B).

### FLS suppress T cell activation induced by macrophages as APCs.

**Figure 5:**
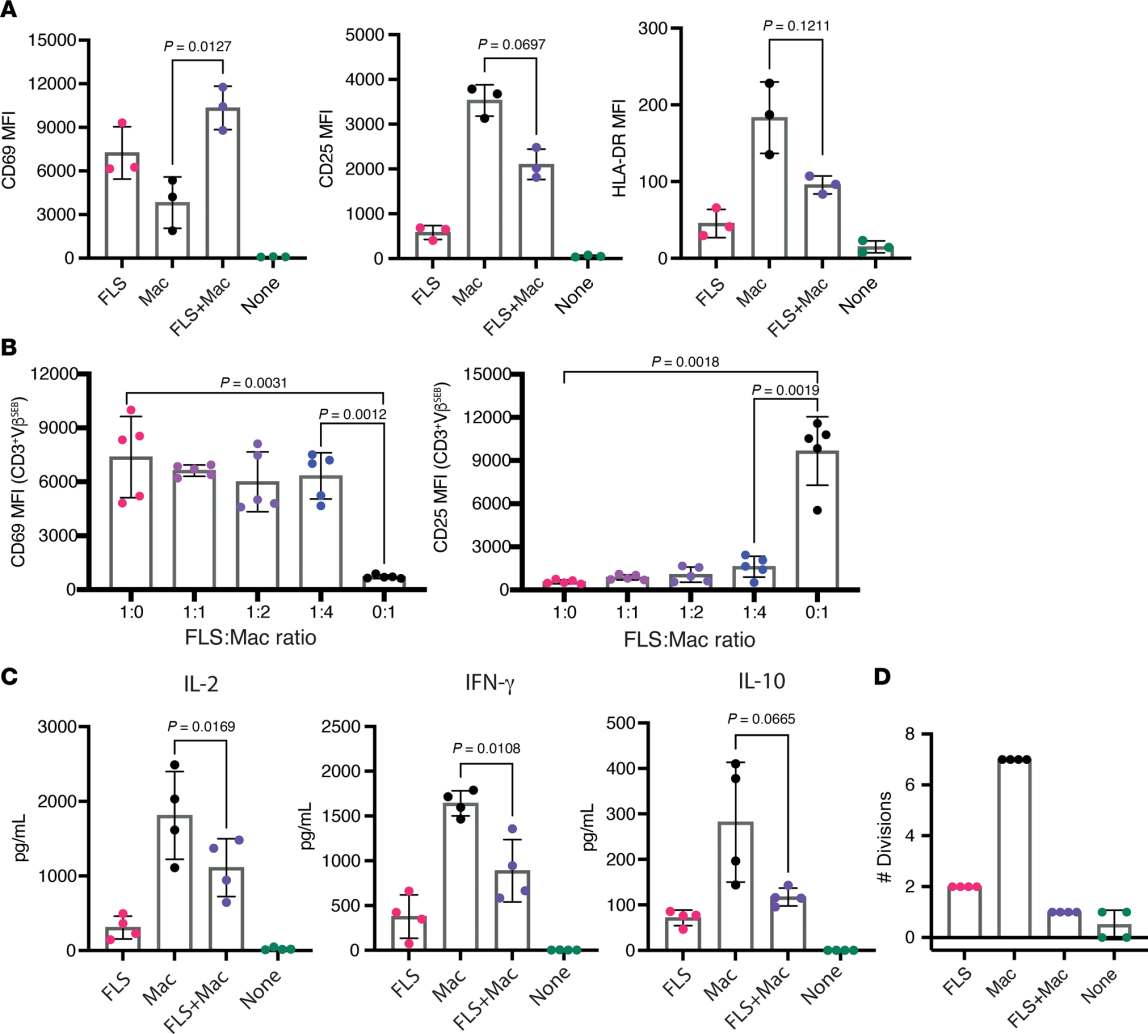
Dominant suppression of macrophage-induced T cell activation by FLS. (**A**) The surface expression of T cell activation markers CD69, CD25, and HLA-DR on CD3^+^ Vβ^SEB^ T cells measured via flow cytometry after 72 hours of SEB activation with APCs as shown. *N* = 3. (**B**) The surface expression of T cell activation markers CD69 and CD25 on CD3^+^ Vβ^SEB^ T cells measured via flow cytometry after 72 hours of SEB activation with the FLS/macrophage ratios shown. *N* = 5. (**C**) Cytokine concentrations measured by Luminex in the supernatant of APC–T cell cocultures with or without SEB stimulation for 48 hours. For **A**–**C**, statistical comparisons made using a paired 2-tailed *t* test. *N* = 3. (**D**) Proliferation of CD3^+^ Vβ^SEB^ T cells after 6 days of SEB stimulation. Maximal number of divisions with ≥5% of CD3^+^ Vβ^SEB^ T cells. *N* = 4. For **A**, **C**, and **D**, magenta: FLS as APC; black: macrophages as APC; purple: FLS and macrophages as APC; teal: no APC. For all panels, mean ± standard deviation is shown, and statistical comparisons were made using paired 2-tailed *t* tests with corrections for multiple comparisons within each outcome in **B** to maintain an overall type I error rate 0.05 as described in the methods section.

As FLS and macrophages coexist within the stromal microenvironment of the synovium, we sought to determine how T cell activation is influenced when both cell types are present to act as APCs. We cultured both FLS and macrophages together prior to adding T cells and SEB. We found that FLS exerted an antagonistic effect on macrophage-induced T cell activation (Figure 5A). Specifically, the presence of FLS reduced macrophage-induced CD25 and HLA-DR expression on the T cell surface. Interestingly, combining FLS and macrophages as APCs led to even higher CD69 expression on the T cell surface than when either APC was used alone. As the relative abundance of FLS and macrophages in the RA synovium likely varies considerably between patients, we cocultured FLS and macrophages at different ratios and found that FLS were able to drive CD69 expression and inhibit CD25 expression in T cells even when outnumbered 4:1 (Figure 5B).

To further validate these observations, we measured cytokine concentrations in the supernatant by a Luminex assay. The addition of FLS to macrophages as APCs decreased T cell secretion of effector cytokines IL-2, IFN-γ, and IL-10 after SEB activation (Figure 5C). Of note, IL-10 is expressed by heterogeneous populations of T cells, including some that coexpress IFN-γ (19), and our data may more generally support a model in which FLS globally dampen T cell cytokine expression. We observed similar patterns when DCs were used as APCs in place of macrophages (Supplemental Figure 6).

Finally, we assessed the impact of FLS and macrophages on T cell proliferation. Cocultures with both FLS and macrophages as APCs led to a marked reduction of T cell proliferation compared with FLS alone, highlighting a compounding inhibitory effect on proliferation (Figure 5D).

### IDO1-driven tryptophan depletion within ectopic lymphoid structures drives FLS effects on T cells.

**Figure 6:**
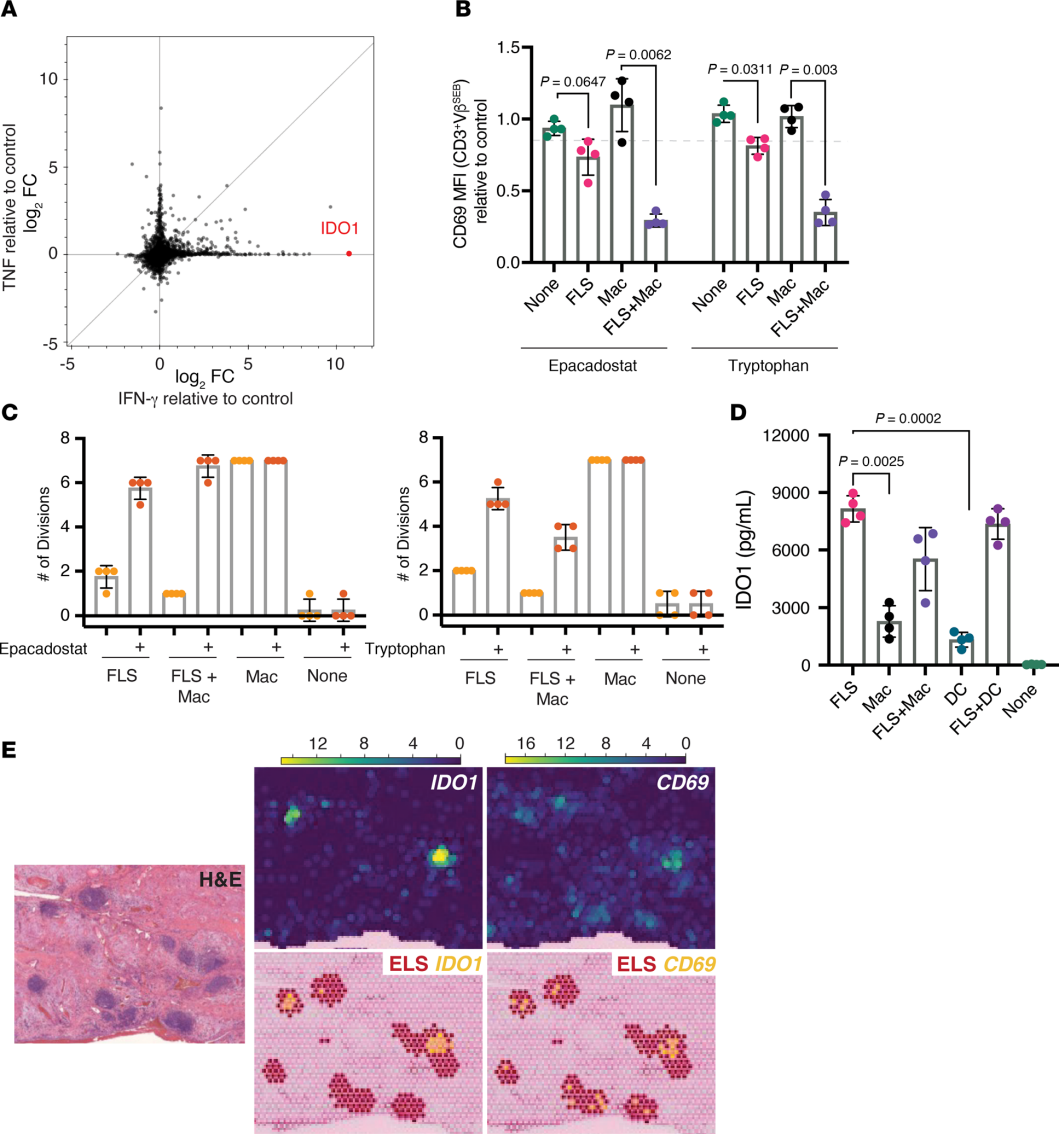
IDO1-induced tryptophan depletion within ectopic lymphoid structures is associated with the FLS effects on T cell activation. (**A**) *IDO1* is the most highly induced gene in FLS stimulated with IFN-γ in vitro. RNA sequencing of FLS stimulated with IFN-γ (50 pg/mL) or TNF (100 pg/mL) for 24 hours relative to an unstimulated control (log_2_ fold change [FC] shown) (4). Genes shown as black dots except *IDO1*, which is highlighted in red. (**B**) IDO1 inhibition (epacadostat) or tryptophan supplementation reduced FLS-induced CD69 expression. CD69 expression (MFI) on CD3^+^ Vβ^SEB^ T cells after 72 hours of SEB stimulation with the APC indicated and treatment with selective IDO1 inhibitor epacadostat 100 nM (left) or tryptophan 250 mM (right) relative to DMSO (for epacadostat) or media (for tryptophan) treated controls. *N* = 4. (**C**) IDO1 inhibition (epacadostat, left) or the addition of tryptophan (right) reduces FLS-induced suppression of T cell proliferation after SEB stimulation for 6 days. Proliferation measured by CellTrace Violet intensity via flow cytometry. Maximal number of divisions with ≥5% of CD3^+^ Vβ^SEB^ T cells are shown. APC indicated below *x* axis. *N* = 4. (**D**) IDO1 is more highly expressed by FLS than macrophages or DCs. IDO1 expression measured by Luminex assay from supernatant of T cell–APC cultures stimulated by SEB for 48 hours. APC identified on *x* axis. *N* = 4. (**E**) *IDO1* and *CD69* expression (spatial transcriptomics) in a representative RA synovial tissue section with multiple ectopic lymphoid structures (ELS). *N* = 8 (22). Top, expression of *IDO1* and *CD69* (color scale: read counts). Bottom, coplotting of RNA capture spots containing ELS (maroon) and *IDO1* or *CD69* (yellow). Left, corresponding H&E. For **B**–**D**, mean ± standard deviation is shown, and statistical comparisons were made using paired 2-tailed *t* tests with corrections for multiple comparisons within each outcome to maintain an overall type I error rate of 0.05 as described in the Methods section.

Prior work identified IFN-γ–dependent tryptophan metabolism as the primary mechanism through which FLS suppress T cell proliferation (13). In this model, IFN-γ–stimulated FLS express indoleamine 2,3-dioxygenase (IDO1), an enzyme that catabolizes tryptophan into kynurenine. This process depletes tryptophan in the culture media, limiting T cell proliferation via activation of the stress response kinase GCN2 (20). To investigate how this mechanism applies to FLS-activated T cells, we first confirmed that *IDO1* is highly expressed by IFN-γ–stimulated FLS (Figure 6A). Among all genes induced by IFN-γ in cultured primary FLS, *IDO1* was the most differentially expressed compared with an unstimulated control.

In agreement with the published model (13), we found that both IDO1 inhibition with a selective inhibitor, epacadostat (21), as well as the addition of excess tryptophan, decreased T cell CD69 expression when FLS were present (Figure 6B). Neither intervention affected CD69 expression in the absence of an APC or when macrophages were the sole APCs. Additionally, the addition of either epacadostat or tryptophan rescued the suppression of T cell proliferation observed with FLS as APCs (Figure 6C).

To further investigate IDO1 expression, we compared IDO1 production across APC–T cell cocultures stimulated with SEB. FLS as APCs induced the highest levels of IDO1 in the culture supernatant compared with monocyte-derived macrophages or DCs (Figure 6D), verifying that IDO1 is primarily produced by FLS rather than professional APCs.

To explore the relevance of this IDO1-dependent mechanism in vivo, we analyzed spatial transcriptomics data from 8 RA synovial tissue sections (22). We hypothesized that IDO1-driven tryptophan depletion in situ would coincide with areas of high CD69 expression on T cells, reflecting FLS-driven T cell activation. In our analysis of RA synovial sections, we were able to detect both *IDO1* and *CD69* expression in all 8 tissue sections and noted that the expression was often, but not exclusively, within well-defined ELS, which have a high concentration of T cells (Figure 6E). Our prior work demonstrated that FLS within ELS express nuclear phosphorylated STAT1, indicative of an IFN-γ response (4), which induces *IDO1* expression in FLS (Figure 6A). We assessed the colocalization of *IDO1* with *CD69* and macrophage/DC markers in a spatial transcriptomics dataset (22), and while the global Lee’s L spatial correlation was not significant for any of the pairwise correlations because of low transcript counts, the local Lee’s L revealed that *IDO1* expression was negatively spatially correlated with *CD14* but strongly positively correlated with that of *CD69* and *LAMP3*. *LAMP3* expression is a marker of mature regulatory DCs, which are known to express *IDO1* (23) and localize to ELS (22) (Supplemental Figure 7). Although the synovial ELS microenvironment appears to support both *IDO1* and *CD69* expression, the limited spatial resolution of the dataset and the high cell density within ELS limits our ability to correlate *CD69* expression on T cells with *IDO1* expression by an APC. However, given the ubiquity of FLS in the synovium as one of the primary resident cell types and the high expression of IDO1 observed by FLS in vitro relative to macrophages or DCs, these findings suggest that FLS-mediated tryptophan depletion, driven by IDO1, may contribute to T cell CD69 expression and suppression within the RA synovium in vivo.

## Discussion

HLA-D–expressing fibroblasts are found in multiple human tissues, where they may present antigens to T cells with functional consequences that vary depending on the tissue type and the specific inflammatory niche. In the lymph node, stromal cells present self-antigens via MHC class II, leading to CD4^+^ T cell tolerization and Treg proliferation (24, 25). In pancreatic adenocarcinoma, antigen-presenting cancer-associated fibroblasts (apCAFs) have an immunosuppressive role, enabling tumor immune evasion (26). Conversely, in non–small cell lung cancer, apCAFs activate and sustain CD4^+^ T cells, promoting antitumor immunity (27). Here, fibroblast-specific ablation of MHC class II impaired local immune responses and accelerated tumor growth (27). It may be that fibroblasts can both activate and suppress T cells and that it is the balance of these 2 functions that determine the overall effect. Here, we demonstrate that antigen presentation by RA FLS results in hyporesponsive T cells, characterized by limited expression of functional activation molecules CD25 (IL-2RA) and HLA-DR, reduced effector cytokine production (IL-2 and IFN-γ), and suppressed proliferation compared with T cells activated by professional APCs. Instead, high expression of T cell CD69 marks the HLA-D/TCR FLS–T cell interaction.

Although CD69 is well known as an early marker of lymphocyte activation, it also has functional roles. CD69 inhibits sphingosine-1 receptor–dependent lymphocyte trafficking out of lymphoid organs (28) and tissues (12), limits pro-inflammatory cytokine production via a TGF-β–dependent mechanism (29), and suppresses Th17 differentiation in favor of Tregs (30). Thus, after its initial role as an activation marker, CD69 primarily functions to suppress immune responses. In RA, infiltrating T cells are predominantly of a memory phenotype and widely express CD69 (16, 17, 31). Consistent with CD69’s function, infiltrating T cells isolated from RA synovial fluid express high levels of CD69 (32), are hyporesponsive to stimulation (32), and persist in the synovium without migrating in response to tissue egress signals (17). As tissue-resident synovial cells, FLS are well positioned to modulate the activation state of the memory T cells that dominate the synovium (Figure 4).

Our data suggest a model where IFN-γ–expressing T cells infiltrating the synovium activate FLS to express surface HLA-D and secrete soluble IDO1. The high levels of IDO1 produced by FLS (compared with professional APCs, Figure 6D) deplete local tryptophan available to interacting T cells (13). Consistent with this model, inhibiting IDO1 with epacadostat or supplementing with excess tryptophan alleviated FLS-induced T cell suppression — both reducing CD69 expression and restoring proliferation (Figure 6, B and C). We propose that the high CD69 expression on T cells contributes to their hyporesponsiveness and inability to traffic out of the joint to regional lymph nodes. Supporting this model, spatial transcriptomic analysis showed colocalized *IDO1* and *CD69* within lymphoid-rich regions of synovial tissues (Figure 6E).

Is FLS HLA-D expression necessary for the immunosuppressive effects on T cells? The most important role of HLA-D expression may be to promote stable physical interactions between FLS and T cells. By effectively retaining T cells in a high-IDO1, low-tryptophan microenvironment, FLS amplify their suppressive effects. Although our model shows direct TCR engagement by HLA-DR^+^ FLS, physical contact is not strictly required for FLS to modulate T cell activation: (i) non-SEB-responsive T cells (Vβ^non^) with FLS as APCs display higher CD69 expression than their counterparts with macrophages as APCs (Figure 1A); (ii) T cell activation with anti-CD3/CD28 beads in the presence of FLS results in higher CD69 expression and reduced proliferation than when activated alone (Figure 2); and (iii) when FLS and a macrophage are cocultured as APCs, the FLS effect is dominant even when macrophages are present in excess of FLS (Figure 5) and driven by the soluble factor IDO1 (Figure 6, B and C). Therefore, FLS modulate T cell activation most effectively when physically interacting but can have local effects on T cells activated by nearby professional APCs. In this context, FLS HLA-D expression may divert T cells away from professional APCs, reinforcing their suppressive program.

Although this FLS-induced T cell suppression appears to be a general property of fibroblasts (Figure 2), its relevance may be limited to inflammatory contexts, such as with lymphocyte-rich synovia, which comprises approximately half of RA synovial samples (16, 33), and therefore may not be relevant for all patients. Furthermore, while our results clearly show differences in T cell phenotype between FLS- and macrophage-driven antigen presentation, the downstream effects of FLS on T cell differentiation remain incompletely defined. Full characterization of FLS effects on specific tissue-resident memory T cell populations, such as T resident memory and T effector memory cells, was not feasible because of limitations in isolating sufficient T cells for functional studies. As CD69 is a marker of tissue-resident memory T cells, it is possible that FLS contribute to this phenotype within the synovium. Differences in transcription factor expression, RORγt and T-bet, as well as cytokine production, CXCL13, with FLS as APCs suggest that FLS may influence T cell fate (Figure 3). However, these effects are likely to greatly depend on the local tissue microenvironment, as evidenced by the differences in T cell CXCL13 expression with FLS alone versus cultured with macrophages and DCs as APCs (Figure 3C), and may be best studied using spatial technologies with single-cell resolution.

In conclusion, FLS have a predominantly immunosuppressive effect on T cell activation compared with professional APCs. As one of the primary tissue-resident cell types, FLS play a crucial role in maintaining the immunologic balance of the synovium in RA and other forms of inflammatory arthritis. As the multiple roles of FLS are further elucidated and they are investigated as a potential therapeutic target, it will be important to design therapeutics that retain their immunosuppressive effects while limiting their tissue-invasive functions.

## Methods

### Sex as a biological variable.

Most RA samples were from women, consistent with the prevalence of RA and the demographics of the FLARE cohort. While sex was not specifically considered as a biological variable in this study, we have not observed functional differences between FLS from men and women with RA. Deidentified peripheral blood from healthy donors was purchased from the New York Blood Center, where donor sex was unknown.

### Human samples.

Synovial tissue and peripheral blood were collected from patients with RA, OA, and PsA, as diagnosed by a rheumatologist based on clinical exam, serologies (rheumatoid factor and anticyclic citrullinated peptide antibodies), and treatment history, enrolled in the Hospital for Special Surgery (HSS) FLARE study of patients undergoing arthroplasty or synovectomy (Perioperative Flare in Rheumatoid Arthritis: Characterization of Clinical and Biological Features, IRB protocol no. 2014-233). All samples were collected in accordance with institutional ethical guidelines, with informed consent obtained from all participants. Samples were deidentified prior to transport to the research laboratory. Synovial tissue was cryopreserved as tissue fragments in CryoStor CS10 (StemCell Technologies, 07959) within 2 hours of collection. The quality of the synovial tissue and grading of the degree of lymphocytic inflammation were assessed by musculoskeletal pathologist evaluation of H&E-stained sections (34).

### FLS isolation.

Synovial tissue samples were disaggregated into a single-cell suspension as described previously (1). Briefly, cryopreserved synovial tissue was thawed at 37°C, minced into small fragments, and digested in RPMI containing 100 μg/mL DNase I (Roche, 10104159001) and 100 μg/mL Liberase TL (Roche, 05401020001) at 37°C on a MACS rotator for 30 minutes. The digested suspension was filtered through a 70 μm nylon strainer (Corning, CLS352350), washed with RPMI, and centrifuged at 500*g* for 4 minutes at 4°C. The resulting single-cell suspension was filtered again through a 70 μm strainer, counted, and plated in αMEM (Life Technologies, 12561-072) supplemented with 10% premium FBS (R&D Systems, S11150), 1% l-glutamine (Gibco, 25030081), and 1% penicillin-streptomycin (Gibco, 15070063). Cells were passaged using TrypLE (Invitrogen, 12605010) and after passage 2 were composed of an FLS monoculture. All experiments used FLS between passages 3 and 5.

### Dermal fibroblast cell line.

Hs27 foreskin fibroblasts (ATCC, CRL-1634) were provided by Thomas Norman (Memorial Sloan Kettering Cancer Center, New York, New York, USA), who purchased them from ATCC. Prior to use in experiments, Hs27 fibroblasts were grown in DMEM (ATCC, 30-2002) supplemented with 10% FBS (VWR, 97068-085).

### Peripheral blood collection and PBMC isolation.

PBMCs were isolated using SepMate tubes (StemCell Technologies, 85450). Blood was diluted 1:1 with Dulbecco’s PBS (DPBS), layered over Lymphocyte Separation Media (Thermo Fisher Scientific, 45-001-750), and centrifuged at 1,200*g* for 10 minutes at room temperature. The PBMC-containing layer was collected, washed with DPBS, and cryopreserved in CryoStor CS10 at 4 × 10^6^ cells/mL.

### CD14^+^ monocyte isolation and differentiation.

CD14^+^ monocytes were isolated from PBMCs using CD14 MicroBeads (Miltenyi Biotec, 130-050-201) according to the manufacturer’s instructions. Briefly, PBMCs were labeled with CD14 MicroBeads in MACS buffer (DPBS supplemented with 0.5% FBS [HyClone, SH30070.03] and 2 mM EDTA) and applied to a MACS LS Column (Miltenyi Biotec, 130-042-401) placed in a magnetic field. CD14^+^ cells were retained in the column and subsequently eluted.

For macrophage differentiation, CD14^+^ monocytes were seeded in 24-well plates at 1 × 10^6^ cells/mL in RPMI 1640 medium (Gibco, 11875093) supplemented with 10% defined FBS and 1% l-glutamine and treated with 50 ng/mL M-CSF (BioLegend, 574804) for 6 days with a media change on day 3. Cells were detached mechanically and plated at the desired concentration for experiments.

For DC differentiation, CD14^+^ monocytes were seeded in 24-well plates at 2.5 × 10^5^ cells/mL in RPMI with 10% FBS and 1% l-glutamine and treated with 100 ng/mL GM-CSF (BioLegend, 572902) and 25 ng/mL IL-4 (PeproTech, 200-04-100) (35) for 6 days with a media change on day 3 prior to removal of differentiation factors for experiments. DCs were never disrupted and used as an APC in the same wells in which they were differentiated. DC differentiation was validated using HLA-DR, CD1C, and CD11C expression.

### T cell isolation.

CD3^+^ T cells were isolated from PBMCs using the Pan T Cell Isolation Kit (Miltenyi Biotec, 130-096-535) following the manufacturer’s protocol. Briefly, PBMCs were labeled with a biotin-antibody cocktail followed by magnetic MicroBeads targeting the labeled, non–T cell population. The cell suspension was applied to a MACS LS Column placed in a magnetic field, and the flow-through containing unlabeled CD3^+^ T cells was collected. Following isolation, T cells were rested for 6–12 hours in a 24-well plate at 4 × 10^6^ to 6 × 10^6^ cells/mL in RPMI supplemented with defined FBS, l-glutamine, and 25 mM HEPES (Corning, 25-060-Cl) before further use.

### Coculture SEB experiments.

Isolated macrophages and fibroblasts were seeded in a 24-well plate at 50,000 cells/well the day before T cells were added to allow for full adherence. For experiments comparing macrophages and DCs, each were seeded at 2.5 × 10^5^ cells/well, and the coculture experiments were carried out in these wells without disturbing the adherent cells. For all experiments, CD3^+^ T cells were plated at 5 × 10^5^ cells/well in a final volume of 600 mL in RPMI 1640 medium supplemented with 10% defined FBS, 1% l-glutamine, 1% penicillin-streptomycin, and 25 mM HEPES. SEB (Millipore Sigma, 324798) was added to appropriate wells to activate T cells at a concentration of 1 ng/mL. SEB was used by specifically trained personnel and subject to a High Hazard Operating Procedure approved by the institutional Environmental Health and Safety office.

Autologous coculture experiments were conducted using RA FLS, macrophages, and T cells, all derived from the same RA patient donor. FLS (from tissue) and macrophages (differentiated from blood-derived CD14^+^ cells) were plated at a density of 40,000 cells/mL, following the protocols described above. Synovial tissue was dissociated into a single-cell suspension as above, and T cells were sorted using CD45 (APC), CD3 (FITC), and DAPI (dead) staining at the Weill Cornell Flow Cytometry Core. Sorted T cells were rested and then added at a density of 0.9 × 10^5^ to 2 × 10^5^ cell/well (depending on yield from tissue) to either macrophage or FLS cultures.

For investigation of the tryptophan metabolism pathway, cocultures were treated with 250 μM l-tryptophan (Sigma-Aldrich, T8941) or 100 nM epacadostat (Selleckchem, S7910).

### T cell proliferation.

Just prior to coculture and SEB activation, CD3^+^ T cells were labeled with CellTrace Violet (Thermo Fisher Scientific, C34557) per the manufacturer’s protocol. Cells (at 1 × 10^6^/mL) were incubated with 5 μM dye in DPBS at 37°C for 20 minutes in the dark. The reaction was quenched with complete RPMI medium, and cells were washed twice with DPBS. Stained cells were then cocultured with T cells for 6 days prior to analysis via flow cytometry.

### Flow cytometry.

Single-cell suspensions were prepared and washed in FACS buffer (PBS, 0.5% FBS, 2 mM EDTA). Dead cells were excluded via staining with Fixable Viability Dye eFluor 780 (Thermo Fisher Scientific, 65-0865-18,1:1,000). Nonspecific staining was blocked using Human TruStain FcX (BioLegend, 422302, 1:100) in combination with True-Stain Monocyte Blocker (BioLegend, 426102, 1:50). Surface staining was performed by incubating cells with antibodies in FACS buffer for 20 minutes at 4°C. For intracellular staining, cells were fixed and permeabilized with the Cytofix/Cytoperm kit (BD Biosciences, 554714) and stained with intracellular antibodies in Perm/Wash buffer. Intracellular staining of Foxp3 as well as transcription factors RORγt and T-bet was performed using the eBioscience Foxp3/Transcription Factor Staining Buffer set (Thermo Fisher Scientific, 00-5523-00) according to the manufacturer’s instructions. After final washes, cells were resuspended in FACS buffer and filtered before flow cytometry. All samples were run on a BD Biosciences FACSymphony A3, except those for Figure 3D and Figure 4C, which were run on a BD Biosciences FACSymphony A5 SE. See Supplemental Table 2 for full list of antibodies used in this study.

### RNA sequencing.

RA FLS, macrophages, and T cells were cultured under the conditions described above. After 48 hours, cells were harvested, pelleted by centrifugation at 1,000*g* for 4 minutes at 4°C, and resuspended in 350 μL of Buffer RLT (QIAGEN, catalog 79216) supplemented with 1% β-mercaptoethanol for RNA preservation. Total RNA was extracted using the RNeasy Micro Kit (QIAGEN, catalog 74004). Prior to library construction using the New England Biolabs Ultra II Directional RNA Library Prep Kit for Illumina, RNA quality was determined by Agilent Bioanalyzer. Libraries were sequenced on an Illumina NovaSeq X Plus (paired end, depth of ~40 million reads per sample) in the Weill Cornell Genomics Resources Core Facility. Data analysis was conducted by the HSS Genomics Core Facility. Reads were mapped against the human genome (hg38) with STAR aligner (36) and Gencode v39 (37). Differential gene expression analysis was performed in R using the edgeR package (38). Genes with low expression levels (<3 counts per million) were filtered from all downstream analyses. The Benjamini-Hochberg FDR procedure was used to calculate FDR. Genes with FDR < 0.05 and log_2_ (fold-change) > 1.5 were considered significant.

### Cytokine concentration measurement.

After 48 hours of culture with or without SEB stimulation, supernatant was collected and frozen for analysis. Prior to analysis samples were thawed, vortexed, and centrifuged at 10,000*g* for 8 minutes to remove particulates. Cytokine levels were measured in duplicate using Luminex assay kits with readout on a MAGPIX instrument (Thermo Fisher Scientific). Cytokine 10-plex kit (Thermo Fisher Scientific, LHC0001M) as well as a custom panel (Thermo Fisher Scientific) were used. Cytokine concentration results were not analyzed further if they fell outside of the standard curve.

### Live cell imaging.

Cells were plated in fibronectin-coated, 24-well, glass-bottom plates (MatTek Corporation, P24G-1.5-13-F). Fibronectin (Sigma-Aldrich, F0895) was diluted in PBS (2.5 μg/cm²) and then incubated in the wells for 45 minutes at room temperature prior to use. CellTrace dyes were used to label cells for imaging: CellTrace Violet (Thermo Fisher Scientific, C34557) for macrophages and FLS and CellTrace Far Red (Thermo Fisher Scientific, C34564) for T cells. FLS and macrophages were plated at a density of 1 × 10^4^ cells/well, and T cells were added at 5 × 10^4^ cells/well. The lower cell density was necessary to allow for cell tracking. Plates were imaged using a ZEISS AxioObserver.Z1 microscope with a 20×/0.8NA objective at 10-minute intervals for 12 hours.

For cell contact analysis, cells were considered interacting if they were within a 10 mm distance of one another. Macrophages/FLS (APCs) were segmented based on thresholding the violet channel. T cells were detected as intensity maxima in the far-red channel. A contact between a specific APC and T cell pair was called when a T cell centroid was within 10 mm of the segmented boundary of an APC. T cells and APC were separately tracked in TrackMate (39). A custom Matlab script then loaded both (T cell and APC) tracking results and counted per APC the number and length of contacts with specific tracked T cells. More specifically an APC track was considered robust and reliable if it lasted for at least 30 frames. For each APC that met this criterion, starting at the first frame, a list of the track identifiers of T cells closer than 10 mm was maintained and incremented at each additional frame it was contacted. When a T cell disappeared from the contact list or when the APC track ended, the contact was added to the final report if it lasted for at least 3 frames. Imaging and analysis were performed by the Molecular Cytology Core at Sloan Kettering Institute.

### Spatial transcriptomics.

Spatial data were generated using Visium CytAssist Spatial Gene Expression platform (10x Genomics) in conjunction with the Integrated Genomics Operation and Molecular Cytology core facilities at the Memorial Sloan Kettering Cancer Center and analyzed using DeepTopics (22). ELS shown are Topic 1 from a reference (22). For coplotting the expression of individual genes (IDO1 or CD69) with the ELS topic, genes were considered expressed if they had at least 5 read counts in an RNA capture spot. To measure IDO1 colocalization with other transcripts across spots, a local Lee’s L statistic was calculated (40).

### Statistics.

All statistical analyses were performed using GraphPad Prism 9/10 software. Comparisons between 2 conditions in which T cells were from the same donor were performed using paired 2-tailed Student’s *t* test or nonparametric Mann-Whitney *U* test, as applicable. To adjust for multiple comparisons while maintaining an overall type I error probability of 0.05, the Holm procedure was used (41). For outcomes in which multiple comparisons are made, adjusted *P* values are reported for individual tests. *P* < 0.05 was considered statistically significant. Each dot indicates an individual donor (T cell or FLS). Data are presented as mean ± standard deviation.

### Study approval.

Tissue samples for this study were collected from patients enrolled in the FLARE cohort at the HSS between 2019 and 2023. This study was approved by the IRB (protocol no. 2014-233). All study participants provided informed consent prior to study participation.

### Data availability.

Values for all data points in graphs are reported in the Supporting Data Values file. Raw and processed bulk RNA-sequencing data are available on Synapse (https://doi.org/10.7303/syn68930961; ID syn68930961). Additional requests should be addressed to the corresponding author.

## Author contributions

MRR designed and conducted experiments, analyzed data, and edited the manuscript. PKP analyzed spatial transcriptomics data and prepared figures. EFD and DR isolated synovial tissue and scored synovial histology slides. SMG was the lead clinician for the patient cohort and identified patients for synovial sample collection. CSL oversaw the computational analysis of the spatial transcriptomics data. AYR and LTD conceived the project, acquired funding, supervised research experiments and data analysis, and edited the manuscript. MHS conceived the project, acquired funding, designed and conducted experiments, supervised research experiments, analyzed data, prepared figures, and wrote the manuscript.

## Funding support

This work is the result of NIH funding, in whole or in part, and is subject to the NIH Public Access Policy. Through acceptance of this federal funding, the NIH has been given a right to make the work publicly available in PubMed Central.

- Rheumatology Research Foundation Scientist Development Award (MHS).
- HSS T32 5T32AR071302-04 (MHS).
- NIH National Institute of Arthritis and Musculoskeletal and Skin Diseases (NIAMS) K08AR082929 (MHS).
- NIH National Institute of General Medical Sciences T32GM152349 (PKP through Weill Cornell/Rockefeller/Sloan Kettering Tri-Institutional MD-PhD Program).
- NIH National Institute of Allergy and Infectious Diseases R01 AI034206-28 (AYR).
- NIH National Human Genome Research Institute U01 HG012103 (CSL, AYR, and LTD).
- NIAMS R01 AI148435 (LTD).
- AYR is an investigator with Howard Hughes Medical Institute and is supported by the Ludwig Center for Cancer Immunotherapy at Memorial Sloan Kettering.
- Molecular Cytology Core at the Sloan Kettering Institute, the core grant (NIH P30 CA008748).
- Integrated Genomics Operation Core at the Sloan Kettering Institute, the NIH National Cancer Institute Cancer Center Support Grant (P30 CA08748), Cycle for Survival, and the Marie-Josée and Henry R. Kravis Center for Molecular Oncology.

## Supplementary Material

Supplemental data

Supplemental tables 1-2

Supporting data values

## Version 1 10/07/2025

In-Press Preview

## Version 2 11/24/2025

Electronic publication
