# Supplementary material for: Rheumatoid arthritis synovial fibroblasts modulate T cell activation
Source: JCI Insight. 2025 Oct 7;10(22):e193054. doi: 10.1172/jci.insight.193054 (PMC12643496; doi:10.1172/jci.insight.193054)
Supplement: Supplemental data [file jciinsight-10-193054-s033.pdf]

Supplementary Figures:

Rheumatoid arthritis synovial fibroblasts modulate T cell activation

Melissa R Romoff[1], Preethi K Periyakoil[2], Edward F DiCarlo[3], Daniel Ramirez[3], Susan M Goodman[4,5], Christina S Leslie[2], Alexander Y Rudensky[6], Laura T Donlin[1,5], and Melanie H Smith[1,4,5]

[1] HSS Research Institute, Hospital for Special Surgery, New York, NY, USA.
[2] Computational and Systems Biology Program, Memorial Sloan Kettering Cancer Center, New York, NY, USA
[3] Department of Pathology and Laboratory Medicine, Hospital for Special Surgery, New York, NY, USA.
[4] Division of Rheumatology, Department of Medicine, Hospital for Special Surgery, New York, NY, USA.
[5] Weill Cornell Medical College, New York, NY, USA.
[6] Howard Hughes Medical Institute and Immunology Program at Sloan Kettering Institute, Ludwig Center for Cancer Immunotherapy, Memorial Sloan Kettering Cancer Center, New York, NY, USA

Corresponding Author:
Melanie H. Smith
535 East 70th Street, New York, NY, 10021
+1-212-224-7960
smithmel@hss.edu

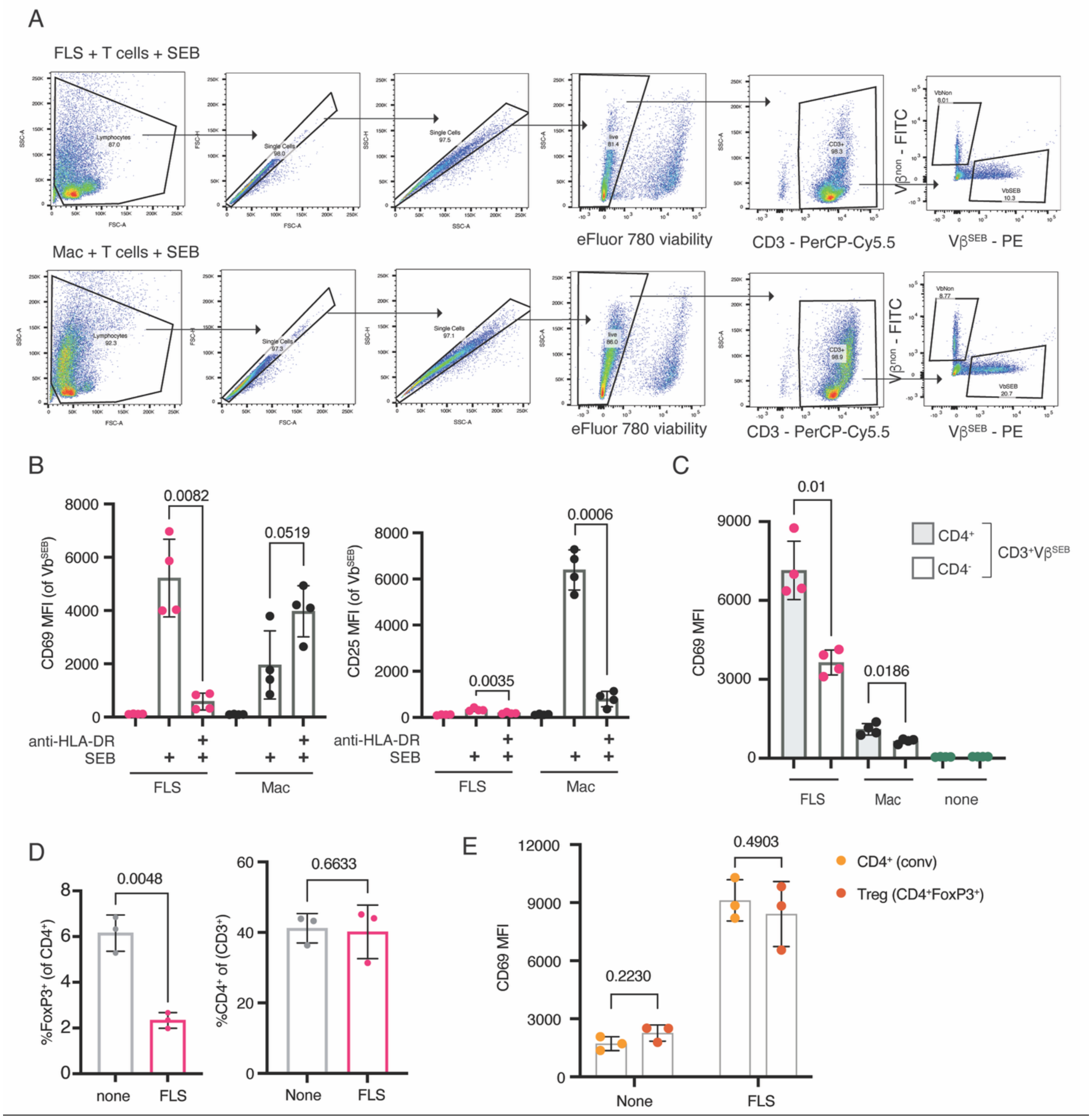

Supplementary Figure 1: FLS-driven CD69 expression on T cells is HLA-DR dependent and predominantly observed in $CD4^+$ conventional T cells. (**A**) Flow cytometry gating of culture condition with FLS or macrophages as APCs and SEB stimulation, illustrating the separation of $V\beta^{SEB}$ and $V\beta^{non}$ T cells. Note differences in T cell side scatter and the percentage of $V\beta^{SEB}$ T cells within the of $CD3^+$ population. (**B**) Addition of HLA-DR blocking antibody (clone L243) inhibited FLS driven CD69 and macrophage driven CD25 expression. N=4. (**C**) CD69 expression (MFI) is higher on $CD3^+Vb^{SEB}CD4^+$ as compared to $CD3^+V\beta^{SEB}CD4^-$ T cells after SEB stimulation for 72 hours. N=4. (**D**) Using anti-CD3/CD28 beads to activate $CD3^+$ T cells either alone (none) or in the presence of FLS (FLS) for 72 hours, $Foxp3^+$ regulatory T cells (Treg) were not expanded in the FLS condition. Percentages of $CD4^+$ (of $CD3^+$) T cells were similar across conditions. N=3. (**E**) In the presence of FLS, CD69 expression (MFI) was the same between conventional $CD4^+$ T cells ($CD3^+CD4^+Foxp3^-$) and Treg ($CD3^+CD4^+Foxp3^+$). N =3. For (**B-E**), mean +/- standard deviation is shown. Statistical comparisons were made using paired two-tailed t-tests with corrections for multiple comparisons within each outcome to maintain an overall Type I error rate of 0.05 as described in the methods section.

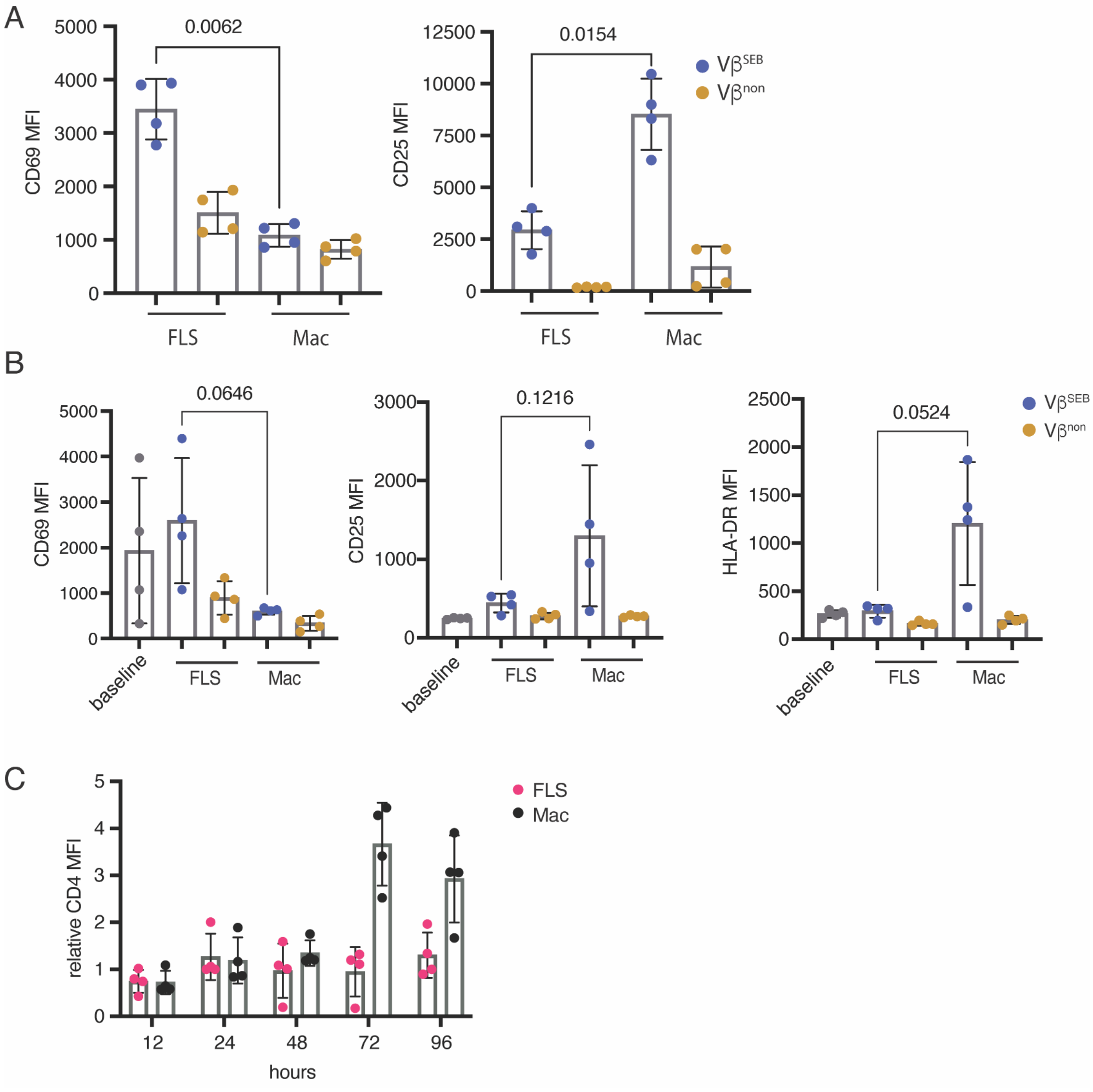

Supplementary Figure 2: FLS effects on T cell activation are the same when compared to autologous synovium-derived T cells and macrophages. (**A**) CD69, CD25 and HLA-DR expression (MFI) on $CD3^+$ with SEB stimulated co-cultures in which both $CD14^+$ macrophages and $CD3^+$ T cells isolated from the RA synovium are used along with autologous FLS. Baseline indicates marker expression prior to co-culture and 72 hour SEB addition. N=4. (**B**) CD69 and CD25 expression (MFI) on $CD3^+$ T cells sorted from RA synovial tissue with a high synovial lymphocytic infiltrate on histologic scoring, cultured with either autologous blood-derived M-CSF differentiated macrophages or FLS, and stimulated with SEB for 72 hours. N = 4. (**C**) Intracellular CD4 expression (MFI) by $CD3^+$ $V\beta^{SEB}$ T cells with FLS or macrophages as APCs relative to no APCs. Magenta: T cells with FLS as APCs; black: T cells with macrophages as APC. N=4. Mean +/- standard deviation is shown. Statistical comparisons were made using paired two-tailed t-tests.

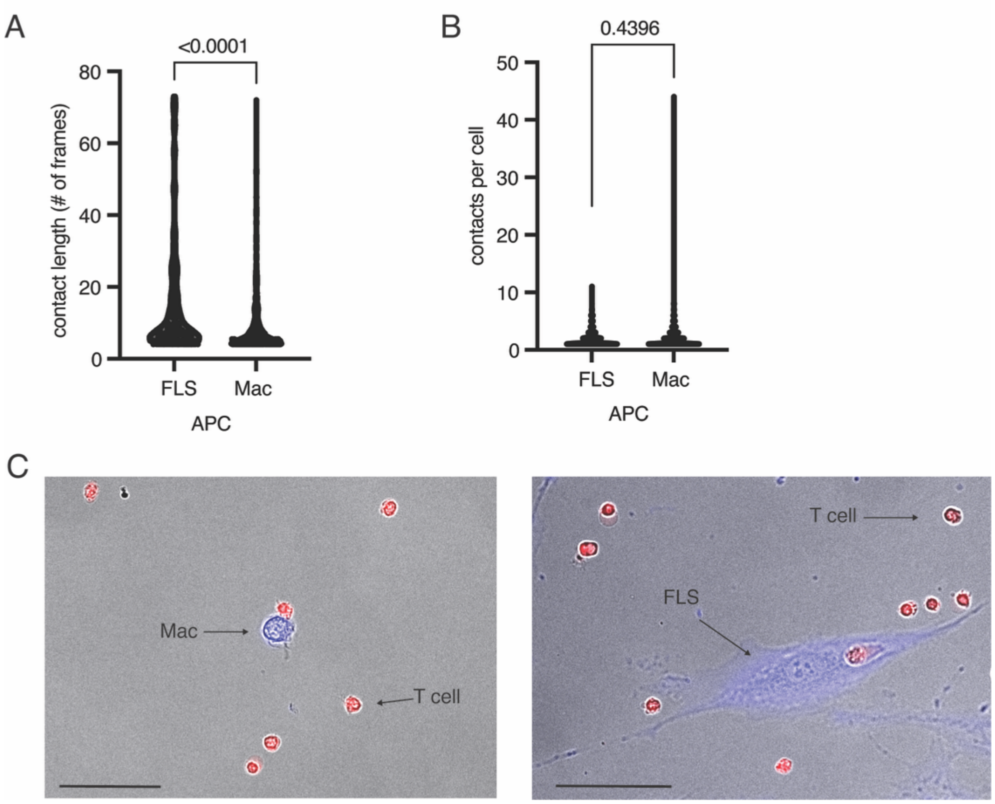

Supplementary Figure 3. **FLS make prolonged contact with T cells.** Using fluorescently labeled APCs (either FLS or macrophages) and T cells (CellTrace dyes) stimulated with SEB, live cells were imaged over 12 hours. Tracked APCs: 1691 FLS (with 215 T cell contacts) and 3713 macrophages (with 735 contacts). (**A**) Contact duration (non-zero, number of frames) between interacting T cells and APCs were measured using a cell area-based analysis with a 10μm distance cut off. (**B**) Number of distinct contacts per APC. For **A** and **B**, statistical comparisons were performed using a two-tailed Mann-Whitney U Test. (**C**) Representative images of macrophages (left) or FLS (right) (both APCs: blue) interacting with T cells (red). Scale bar = 40μm.

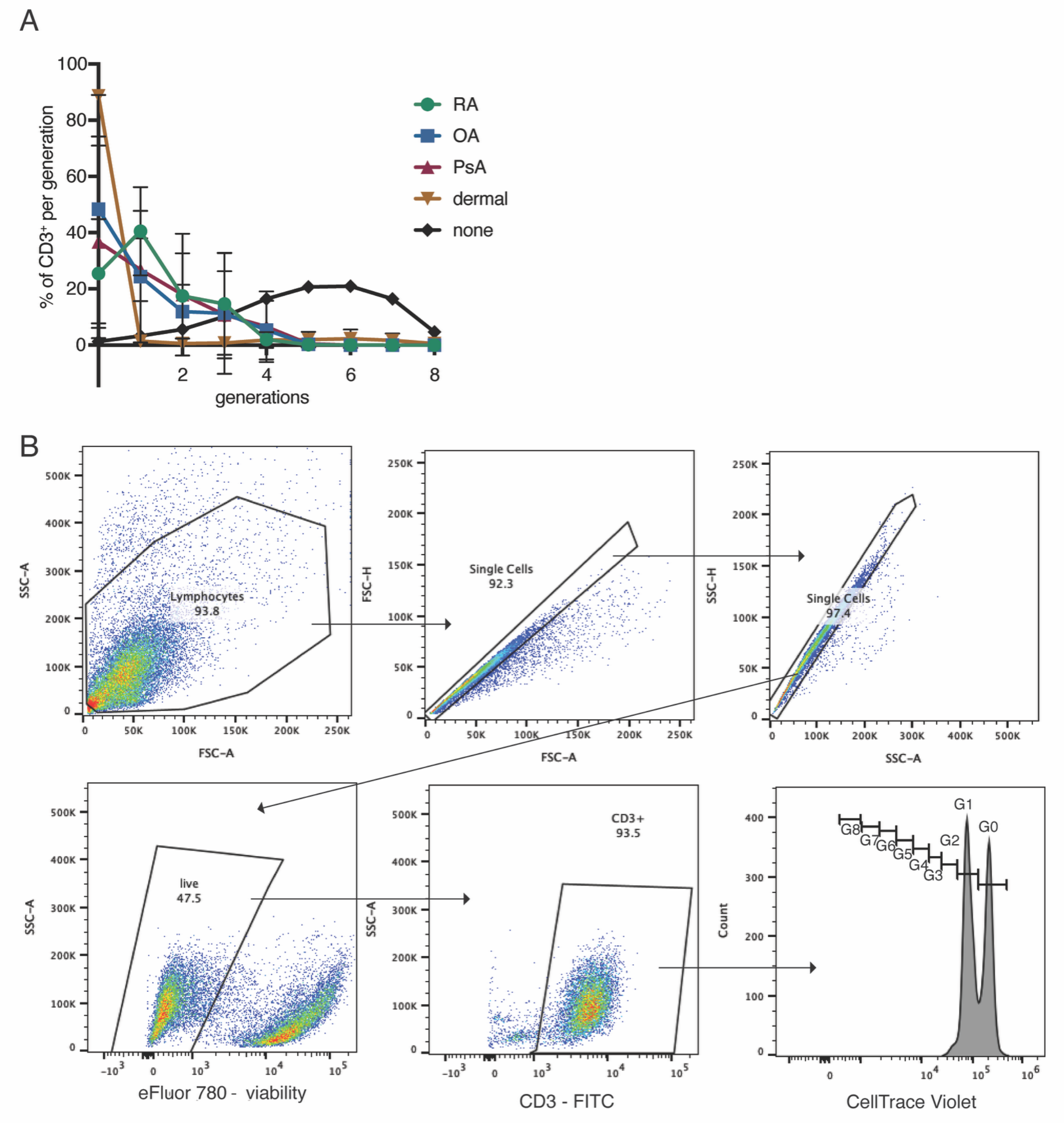

Supplementary Figure 4. FLS from multiple diseases suppress T cell proliferation. (**A**) Proliferation of CD3+ T cells after 6 days of anti-CD3/CD28 bead stimulation with fibroblasts as in Figure 2, in addition to a no fibroblast control (none). Percent of CD3+ T cells in each of 8 observed generations (divisions). (**B**) Gating strategy shown for T cells after bead stimulation in the presence of one of the RA primary FLS donors. Generations labeled G0-G8.

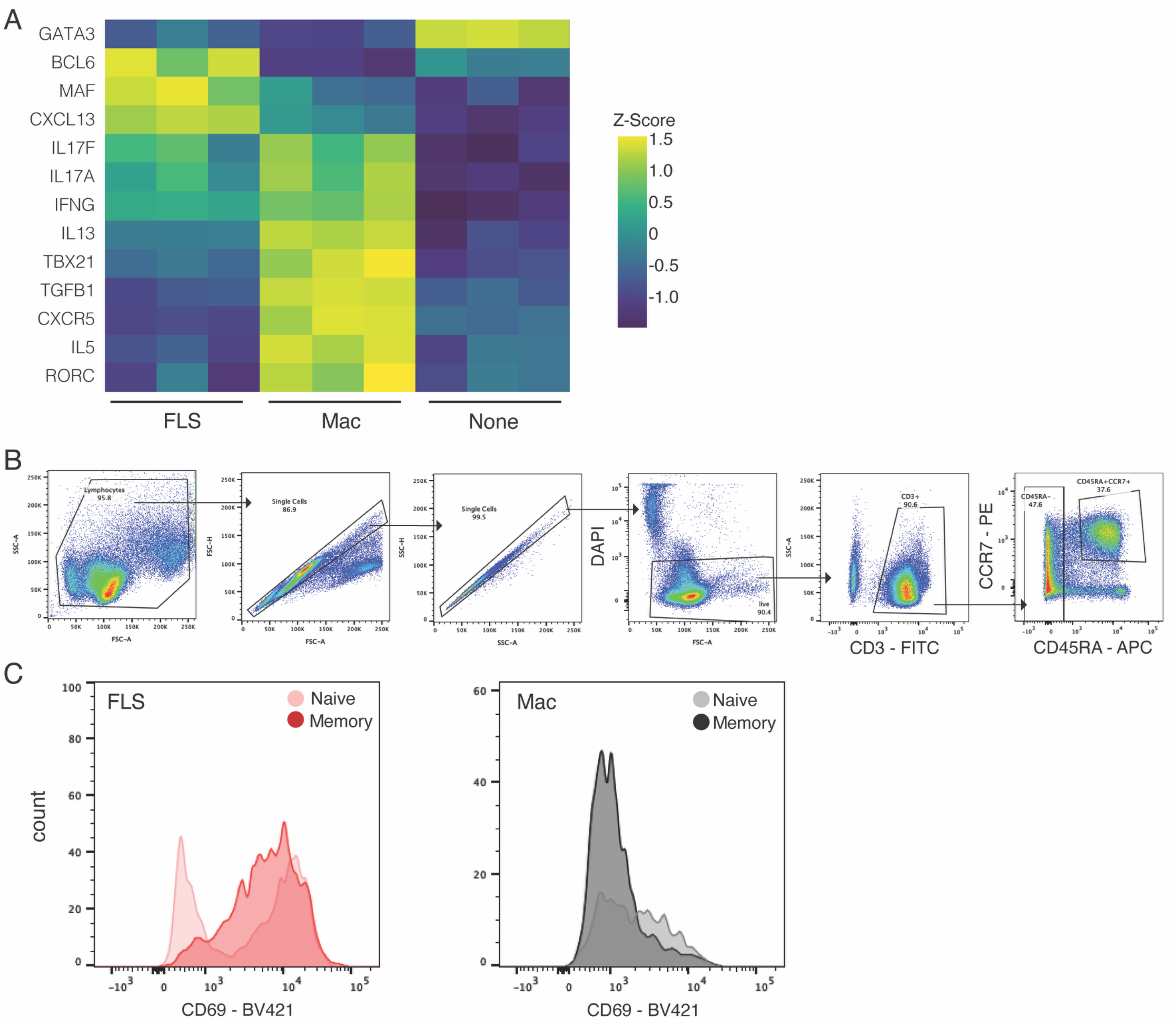

Supplementary Figure 5. APC effect on the expression of T cell expression differentiation factors and memory versus naïve T cells. (**A**) Heatmap of predominantly T cell-expressed genes related to T cell differentiation across conditions (FLS, macrophages, none). N=3 T cell donors for each condition. (**B**) Gating strategy for sorting naïve ($CD45RA^{+}CCR7^{+}$) and memory ($CD45RA^{-}$) $CD3^{+}$ T cells from healthy donor PBMCs. Population percentages shown with gate names are the percent of the parent population. (**C**) Representative histograms from flow cytometric expression of CD69 on $CD3^{+}V\beta^{SEB}CD69^{+}$ cells after 72 hours of SEB stimulation for FLS (left) and Mac (right) as APC from experiment quantified in Figure 4A.

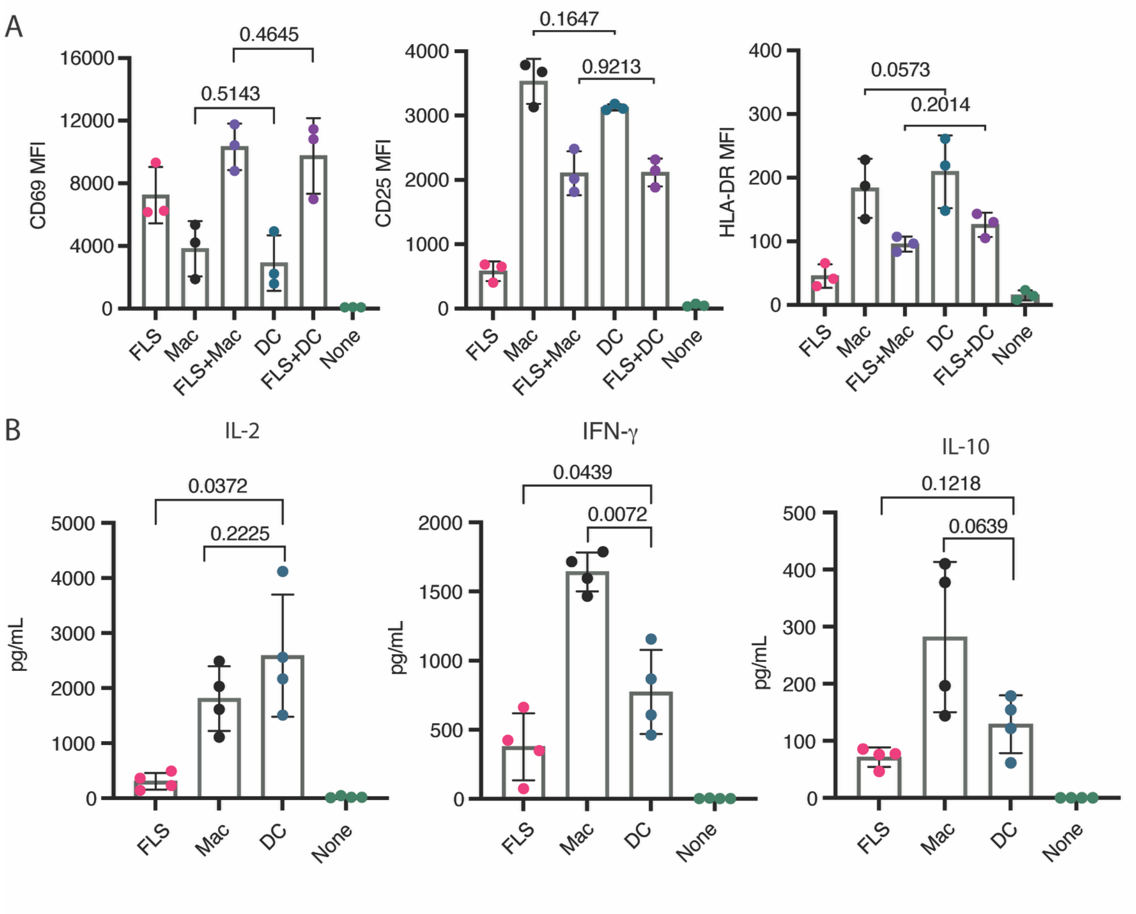

Supplementary Figure 6. **Dendritic cell activation of T cells is similar to that of macrophages**. (**A**) Surface expression (MFI) of activation markers CD69, CD25 and HLA-DR measured on $CD3^{+}V\beta^{SEB}$ T cells via flow cytometry after 72 hours of SEB activation. The APC identity is shown on the x axis. Dendritic cells (DC) were differentiated using GM-CSF and IL-4 from blood-derived $CD14^{+}$. N=3. (**B**) Cytokine concentrations measured by Luminex assay in the supernatant of APC–T cell co-cultures stimulated with SEB for 48 hours. APC identity indicated on the x axis. N=4. Mean +/- standard deviation is shown. Statistical comparisons were made using paired two-tailed t-tests with corrections for multiple comparisons within each outcome to maintain an overall Type I error rate of 0.05 as described in the methods section.

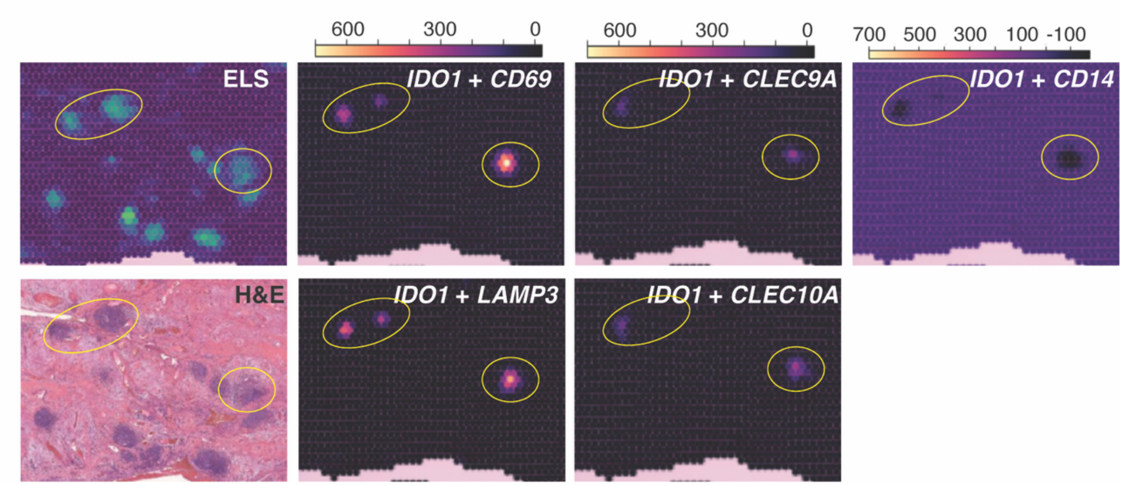

Supplementary Figure 7. Spatial colocalization of *IDO1* with *CD69* and macrophage/DC markers. Expression colocalization across RNA capture spots via local Lee's L statistic. Yellow ovals indicate regions of interest across panels.
